# Combining Transcranial Direct Current Stimulation and Tailor-Made Notched Music Training to Decrease Tinnitus-Related Distress – A Pilot Study

**DOI:** 10.1371/journal.pone.0089904

**Published:** 2014-02-25

**Authors:** Henning Teismann, Andreas Wollbrink, Hidehiko Okamoto, Gottfried Schlaug, Claudia Rudack, Christo Pantev

**Affiliations:** 1 Institute for Biomagnetism and Biosignalanalysis, University Hospital, Münster, Germany; 2 Institute for Epidemiology and Social Medicine, University Hospital, Münster, Germany; 3 Department of Integrative Physiology, National Institute for Physiological Sciences, Okazaki, Japan; 4 Department of Neurology, Beth Israel Deaconess Medical Center and Harvard Medical School, Boston, Massachusetts, United States of America; 5 Department of Otorhinolaryngology, University Hospital, Münster, Germany; University Medical Center Goettingen, Germany

## Abstract

The central auditory system has a crucial role in tinnitus generation and maintenance. Curative treatments for tinnitus do not yet exist. However, recent attempts in the therapeutic application of both acoustic stimulation/training procedures and electric/magnetic brain stimulation techniques have yielded promising results. Here, for the first time we combined tailor-made notched music training (TMNMT) with transcranial direct current stimulation (tDCS) in an effort to modulate TMNMT efficacy in the treatment of 32 patients with tonal tinnitus and without severe hearing loss. TMNMT is characterized by regular listening to so-called notched music, which is generated by digitally removing the frequency band of one octave width centered at the individual tinnitus frequency. TMNMT was applied for 10 subsequent days (2.5 hours of daily treatment). During the initial 5 days of treatment and the initial 30 minutes of TMNMT sessions, tDCS (current strength: 2 mA; anodal (N = 10) vs. cathodal (N = 11) vs. sham (N = 11) groups) was applied simultaneously. The active electrode was placed on the head surface over left auditory cortex; the reference electrode was put over right supra-orbital cortex. To evaluate treatment outcome, tinnitus-related distress and perceived tinnitus loudness were assessed using standardized tinnitus questionnaires and a visual analogue scale. The results showed a significant treatment effect reflected in the Tinnitus Handicap Questionnaire that was largest after 5 days of treatment. This effect remained significant at the end of follow-up 31 days after treatment cessation. Crucially, tDCS did not significantly modulate treatment efficacy - it did not make a difference whether anodal, cathodal, or sham tDCS was applied. Possible explanations for the findings and functional modifications of the experimental design for future studies (e.g. the selection of control conditions) are discussed.

## Introduction

Chronic tinnitus (i.e. permanent and lasting ringing sensation in the ear(s) in the absence of a physical sound source) is a significant public health concern that impairs the quality of life for millions of patients around the world. Tinnitus incidence and prevalence rates appear to be increasing not only in older people, but also in younger adults, probably due to the exposure to occupational and recreational sounds such as amplified music [1], [2].

In the majority of cases, tinnitus is probably triggered by inner ear hair cell injury. Nonetheless, the neural generators of tinnitus are most likely located in the *central* auditory pathway. One possible consequence of injury to hair cells (and the subsequently decreased input to tonotopic maps in auditory cortex) is a loss of lateral inhibition from cortical neural populations which would normally code activity from the now damaged and silent receptors. As a result of such and other disturbances of the balance of excitatory and inhibitory neural transmissions, activations of neural plasticity in the central auditory system lead to alterations of neuronal activity. Among them are (i) hyperactivity, (ii) increased synchrony, and (iii) increased burst firing [3], [4]. Moreover, in many if not all cases of chronic tinnitus, also non-auditory brain structures are part of the tinnitus generating and tinnitus sustaining networks [5].

Traditional and also the more recently developed tinnitus treatment programs use management strategies like cognitive behavior therapy or sound masking which are aimed at the successful habituation of the effects caused by the tinnitus. However, most patients with tinnitus want more of a relief or a cure [6], [7]. Unfortunately, chronic tinnitus has proven to be difficult to treat - presently, there are no curative treatments [1]. One important problem is that chronic tinnitus is most likely a systemic disorder, affecting different parts of the auditory system and other related systems [8]. Another problem is that there are many different treatment targets in the tinnitus network (e.g. auditory cortex, thalamus, dorsal/ventral cochlear nuclei, inferior colliculus, cochlear nerve, and the limbic system [9]). Among those different targets, the auditory cortex might be the most important one, since alterations in its excitatory/inhibitory networks seem to correlate with the subjective tinnitus percept [10]. A non-invasive means to modulate the activity of auditory cortical neural populations contributing to tinnitus perception is acoustic input. Acoustic neuromodulation can be precise and specific by targeting defined auditory neural populations through passive sound stimulation [11] or auditory training [12] using the natural sensory pathway. A recent acoustic neuromodulation strategy is the “tailor-made notched music training (TMNMT)” for chronic tonal tinnitus [13]. TMNMT uses enjoyable, individually modified acoustic input (i.e. patient-selected music notched to exclude the individual tinnitus frequency) to specifically target auditory cortex neuronal populations which code the tinnitus frequency. Both long-term (12 months) and short-term (5 days) TMNMT studies [14]–[16] have yielded results which indicate that TMNMT is a specific treatment holding the potential to reduce tinnitus-related cortical activity along with perceived tinnitus loudness (measured by visual analogue scale) and distress. At this stage of research, it is assumed that TMNMT is suitable for patients with tinnitus frequencies below approximately 8 kHz and without severe hearing losses. We presume that notching out the specific tinnitus frequency (and surrounding frequencies) may confer added benefit compared to other, reportedly effective acoustic neurostimulation approaches, which for instance use complex sounds covering the tinnitus frequency [17], or sequences of pure tones with a distance of one or two octaves to the tinnitus frequency [18]. We assume that the beneficial effects of TMNMT are due to the de-synchronization of tinnitus-related neural activity by lateral inhibition distributed into the notched region [19], [20]. However, another possibility is that listening to notched music for extended periods might rescale auditory sensitivity, leading to a reduction of both the perceived loudness of sound in the notched frequency region and corresponding brain activity [13].

Other forms of non-invasive brain-stimulation have also been used to influence perceived tinnitus loudness and/or tinnitus-related distress. Methods such as transcranial magnetic stimulation (TMS) and transcranial direct current stimulation (tDCS) become increasingly popular to examine causal contributions of particular neural structures to defined cognitive processes (e.g. perception, working memory, or attention) and as neuromodulatory tools to treat patients with psychiatric or neurologic diseases (e.g. depression, schizophrenia, obsessive-compulsive disorder, stroke, Parkinson’s, epilepsy, neuropathic pain, or dysphagia) [21]. Both TMS and tDCS have almost no side-effects if the limits of safe stimulation are met. Compared to TMS, tDCS has the advantages that (i) it does not generate any acoustic noise, and that (ii) effective sham stimulation can be delivered [22]. However, tDCS has the disadvantage of being comparably less focal; the current flow between the cathodal and anodal scalp surface electrodes (i.e. the exact “path” that the current takes through the brain) is not always easy to predict or model [23]. A considerable amount of current can also be shunted through the skin and subcutaneous tissue and does not enter the brain [24]; however, various studies have shown that physiological processes in the brain can be altered by tDCS [25]. Several studies have shown that tDCS does not directly trigger action potentials; rather, neuronal excitability and activity are modulated by tonic de- or hyper-polarization of the resting membrane potential. Thereby, spontaneous neural activity is indirectly manipulated. As a function of stimulation polarity, tDCS can either up- or down-regulate cortical excitability - anodal stimulation leads to cortical excitability increment, while cathodal tDCS causes a decrement [26].

On grounds of studies in animals, it has been proposed that three neuronal mechanisms might underlie tinnitus perception: (i) spontaneous firing rate alterations of central auditory system neurons, (ii) changes in temporal activity patterns of such neurons (increased synchrony), and (iii) plastic reorganization of tonotopic maps [27]. In accordance with this assumption, several [^15^O]H_2_O PET studies [28]–[34] have yielded results indicating tinnitus-related elevated blood flow in auditory structures. Noteworthy, studies using [^18^F]deoxyglucose PET found increased *left* auditory cortex activation in tinnitus patients compared to controls, independent of perceived tinnitus laterality [35]–[37]. Based on these findings, the treatment potential of tDCS over left temporo-parietal cortex has been explored in patients with chronic tinnitus [38], [39]. In both studies, single sessions of anodal or cathodal tDCS were applied. The reference electrode was placed over the right supra-orbital area. Both studies reported significant, transient reductions in perceived tinnitus intensity ([38]: rating scale; [39]: visual analogue scale) under anodal tDCS; no effects were found under cathodal stimulation. Evidently, these effects persisted for several days in some patients [39]. Noteworthy, these findings are counter-intuitive, because anodal tDCS is assumed to increase cortical excitability. So far, there are no studies with repeated applications of tDCS over auditory brain areas in tinnitus patients [40].

However, even though the auditory cortex appears to be an obvious treatment target in tinnitus, it should be noted that the dorsolateral prefrontal cortex (DLPFC) has also been postulated as a possible target for non-invasive brain stimulation, considering that it is important for the integration of sensory and emotional aspects of tinnitus [41]. tDCS over DLPFC was successful in reducing depression, impulsiveness, and pain [42], and some studies have shown that bifrontal tDCS is also effective in alleviating perceived tinnitus intensity (measured by rating scales) and/or perceived tinnitus-related distress (measured by tinnitus questionnaires) to some degree [41]–[46]. Presumably, perceived tinnitus intensity/distress could be modulated directly by targeting both auditory and/or frontal cortices [43]; however, tDCS could also indirectly influence functionally connected brain areas relevant for tinnitus distress and tinnitus intensity [44].

In the recent past, it has become more and more obvious that tinnitus is a system-wide problem [8], which is sustained by a complex and wide-spread tinnitus network [47]. The complexity of the phenomenon needs to be considered when it comes to the development and application of treatment approaches. While effective systemic treatments are not yet available, it appears promising to combine established neuromodulation strategies in order to possibly achieve additive effects. In the present study, we combined two complementary neuromodulation strategies in an explorative manner: (i) TMNMT, and (ii) tDCS over the left auditory cortex. Previous studies [14], [16] have shown that TMNMT is able to specifically reduce potentially tinnitus-related auditory cortex activity, possibly through the activation of neural plasticity; it is assumed that TMNMT attracts lateral inhibition to auditory neurons coding the tinnitus frequency. tDCS, on the other hand, has the potential to either up- or down-regulate neuronal activity and possibly promote plastic reorganization by simultaneously combining tDCS with another sensory stimulation technique [48]–[51]. Moreover, previous studies in healthy subjects have shown that tDCS over auditory cortex is able to modulate both auditory evoked potentials [52] and auditory perception [53]. Furthermore, initial studies in patients demonstrated that tDCS over left auditory cortex *alone* could alleviate perceived tinnitus intensity [38], [39]. Thus, both anodal and cathodal tDCS could theoretically reinforce or facilitate effects of TMNMT. However, due to potentially complex interactions between tDCS polarity and effects induced by the TMNMT treatment sounds, it is hard to predict if and how anodal and/or cathodal tDCS combined with TMNMT would shape perceived tinnitus loudness/distress.

**Figure 1:**
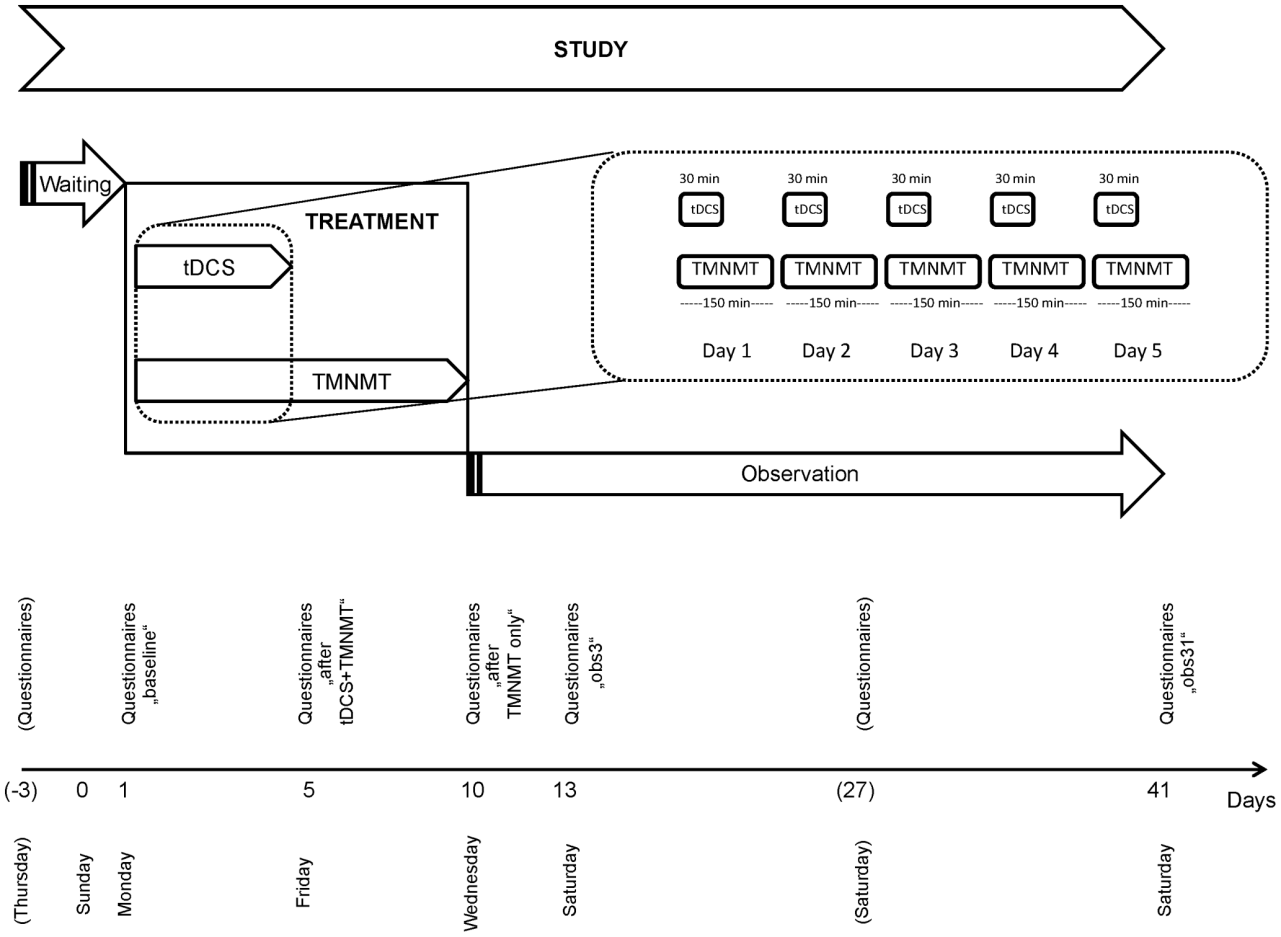
Study design. For each participant, the study took 45 days (4 days of pre-treatment waiting, 10 days of treatment, and 31 days of post-treatment observation). During the initial 5 days of treatment, transcranial direct current stimulation (tDCS) and the tailor-made notched music training (TMNMT) were applied simultaneously; during the remaining 5 days of treatment, only TMNMT was applied. Throughout the study, perceived tinnitus-related distress data were sampled repeatedly.

Based on these considerations, we investigated whether tDCS polarity over left auditory cortex would *modulate* the efficacy of short-term combined tDCS + TMNMT treatment for not severely hearing impaired patients suffering from chronic tonal tinnitus. TMNMT (2.5 hours of training per day over 10 subsequent days) and tDCS (30 min of – depending on treatment group membership - either anodal (N = 10), cathodal (N = 11), or sham (N = 11) stimulation) were applied simultaneously - the direct current was delivered while the patients were listening to their individually modified training music. Given that TMNMT is a re-training strategy, requiring repeated and regular “exercise”, we decided to also apply tDCS repeatedly (5 subsequent days) (Figure 1). Iterative tDCS appears promising also against the background of lasting stimulation after effects, which seem to represent transient modulations of synaptic transmission efficacy [22], and which might permit effect accumulation.

## Materials and Methods

### Ethics Statement

The study was performed in accordance with the Declaration of Helsinki and was approved by the Ethics Commission of the Medical Faculty, University of Münster, Germany. The patients gave written informed consent for the participation in the study.

### Participants

We recruited 34 patients who had (i) chronic (≥3 months) tonal tinnitus, (ii) dominant tinnitus frequencies below 9 kHz, and (iii) reported to have no history of psychiatric or neurologic diseases. All patients reported hearing one single tinnitus percept. Tinnitus could be either uni- or bilateral. In case of bilateral tinnitus, the dominant tinnitus frequency did not differ between ears according to patient’s reports.

**Figure 2:**
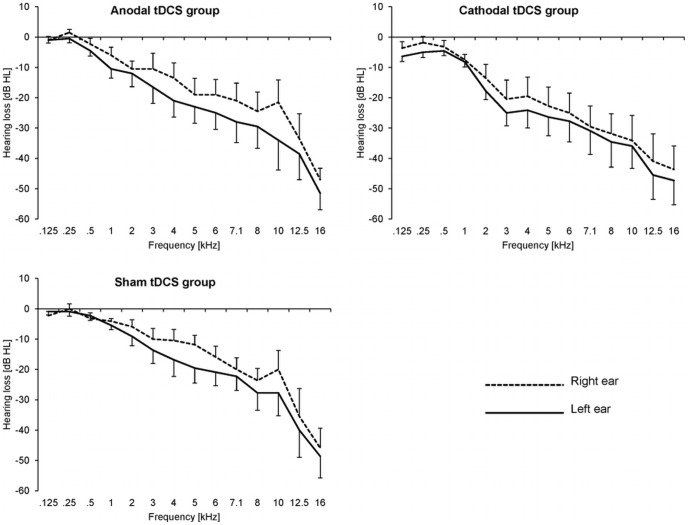
Average hearing thresholds. Thresholds from 0.125 to 16(tDCS) condition (anodal group vs. cathodal group vs. sham group) and ear (left vs. right). The error bars denote standard error of the mean. Negative values reflect hearing loss.

Two patients dropped out during treatment (patient 1 (cathodal tDCS treatment) due to an inability to comply with the study requirements; patient 2 (anodal tDCS treatment) due to novel tinnitus percepts arising in the treatment phase). 32 patients (94.1%) completed the study. Table 1 displays average patient characteristics. Figure 2 displays average hearing thresholds of the patients.

**Table 1:** Patient characteristics.

Groups	Age^1^[years]	Tinnitusfrequency^2^ [Hz]	Tinnitusduration^1^ [years]	General psychopathologicaldistress^1^ [SCL-90-R^3^]	Depression^1^[ADS-L^4^]	State anxiety^1^[STAI^5^]
**Anodal tDCS** 6 **(N = 10)**	42.90 (6.87)	4440.28 (1.78)	10.70 (7.26)	36.90 (39.84)	8.00 (5.85)	36.50 (11.37)
**Cathodal tDCS** **(N = 11)**	44.45 (13.29)	4654.66 (1.34)	10.27 (11.33)	30.91 (25.11)	12.09 (7.46)	35.82 (8.53)
**Sham tDCS (N = 11)**	44.91 (9.92)	4119.98 (1.69)	5.82 (6.15)	28.27 (25.75)	7.45 (6.67)	37.18 (10.45)

1 Arithmetic mean (standard deviation).

2 Geometric mean (standard deviation in octaves).

3 Symptom Checklist 90 Revisited [57];

4 Allgemeine Depressionsskala, Langform [58];

5 State-Trait Anxiety Inventory [59].

6 Transcranial direct current stimulation.

### Study Design

The participants were randomly assigned to one out of three tDCS treatment conditions: (i) anodal group (N = 10), (ii) cathodal group (N = 11), (iii) or sham group (N = 11). Retrospectively, there were no significant differences between groups regarding relevant patient characteristics (cf. Table 1 and section “Patient characteristics” below). All patients received a combined tDCS + TMNMT treatment. The study was performed double-blindly. Prior to the study, the patients were informed that they would receive target (i.e. anodal or cathodal) tDCS treatment with a likelihood of 66.67%, and placebo (i.e. sham) tDCS treatment with a likelihood of 33.33%. Moreover, in order to reduce potential unspecific treatment effects, the patients were also told that they would receive target or placebo TMNMT with a likelihood of 50%. In fact, all patients were treated with target TMNMT; placebo TMNMT was not administered [16]. After the study, the patients were de-briefed.

The treatment phase took 10 subsequent days (1–10; Monday to Wednesday). The tDCS treatment was administered for 5 consecutive days (1–5; Monday to Friday); the TMNMT was administered for 10 consecutive days (1–10; Monday to Wednesday). During the initial 5 days (1–5; Monday to Friday), both treatments were administered simultaneously. In this phase, the patients received tDCS during the initial 30 min of music listening (which took 2.5 hours per day without interruptions). During the last 5 days (6–10; Saturday to Wednesday), only the TMNMT was administered. A waiting phase of 4 days (-3 to 0; Thursday to Sunday) preceded treatment onset. Treatment offset was followed by an observation phase of 31 days (11–41; Thursday to Saturday) (Figure 1).

### tDCS Specifics

tDCS was applied using the “DC-STIMULATOR PLUS” (neuroConn GmbH, Ilmenau, Germany). Independent of tinnitus laterality, the active electrode (area = 35 cm^2^) was horizontally placed over the skull surface representation of left Heschl’s Gyrus (1 cm inferior to the halfway point between C3 and T3 of the 10–20 system of EEG [53]). The reference electrode (area = 100 cm^2^) was placed contra-laterally to the active electrode in the supra-orbital region, just above the right eyebrow. The current strength was set to 2 mA. Stimulation duration was 30 min per training day.

**Figure 3:**
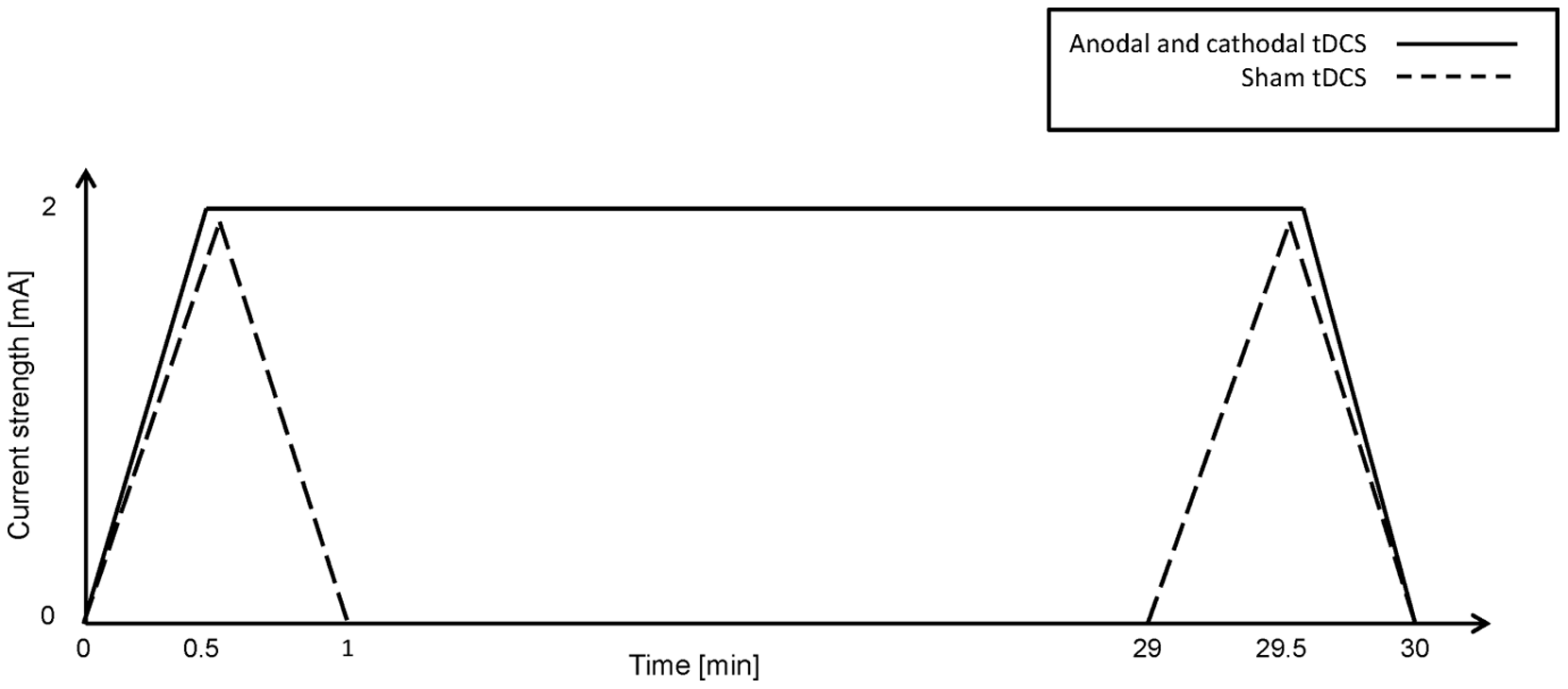
Transcranial direct current stimulation (tDCS). Time course of direct current strength in the different tDCS conditions.

In the anodal and cathodal (i.e. the “real”) tDCS conditions, the direct current was faded in to 2 mA over the course of 30 sec. After 29.5 min of stimulation, the current was faded out to 0 mA. Total stimulation duration was 30 min. In the sham tDCS condition, the direct current was faded in to 2.0 mA and then directly faded out to 0 mA, in each case over the course of 30 sec. The same procedure was repeated 29 min after stimulation onset (Figure 3). Thus, in both the “real” and the sham stimulation conditions, the patients felt the tingling sensation of stimulation, but in the sham condition basically no current was delivered for the duration ( = 30 min) of the “stimulation” session.

### TMNMT Specifics

**Figure 4:**
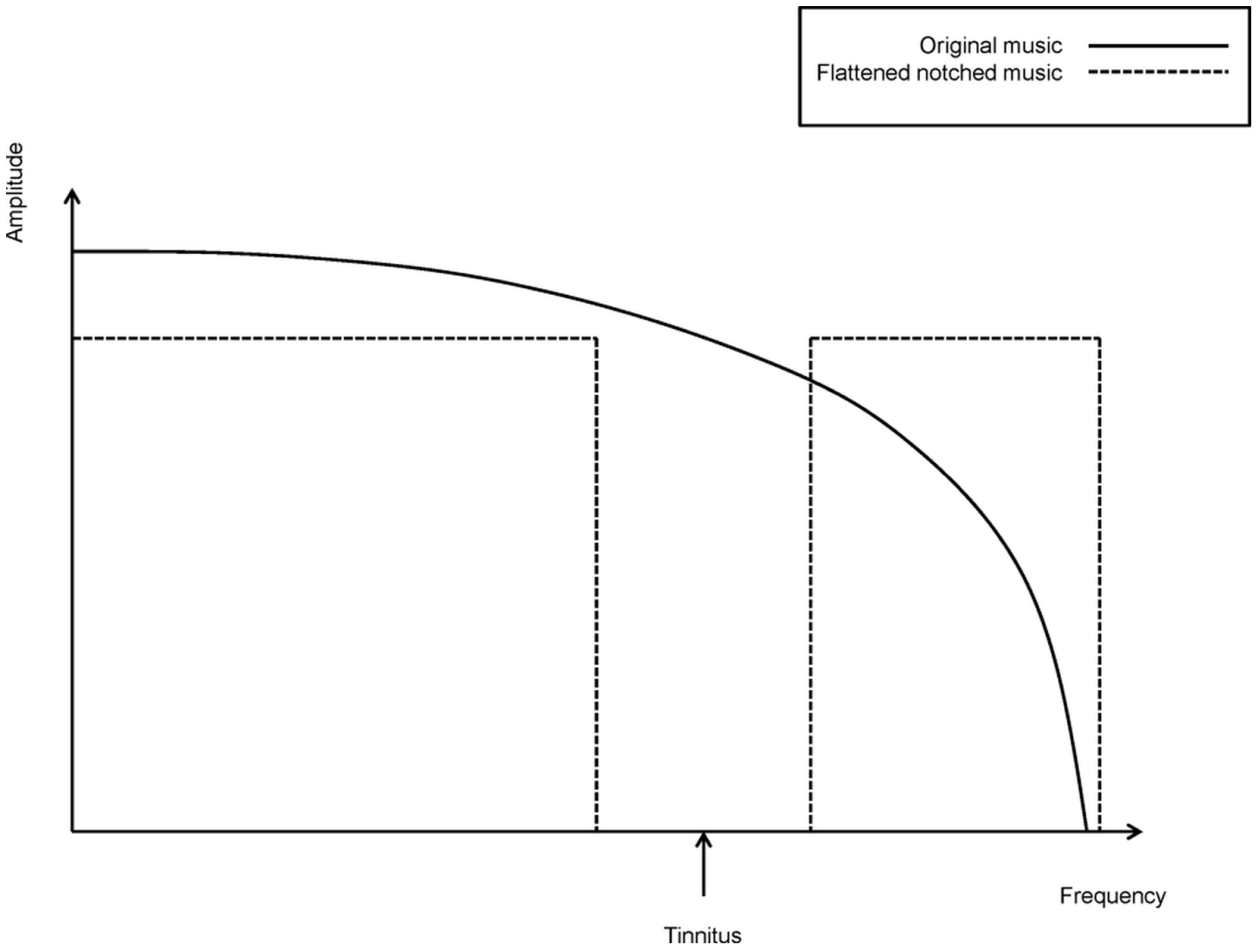
Music spectra. Schematic frequency spectra of original music (solid line) and flattened notched music (dashed dotted line).

Each patient provided up to 10 hours of their favorite music in CD quality (44100 Hz, 16 bit, stereo). The music was modified in two successive steps. First, the energy spectrum of the music was “flattened” by the re-distribution of energy from low to high frequency ranges. Second, the frequency band of one octave width centered at the individual tinnitus frequency (i.e. the most prominent pitch match frequency) was removed from the music energy spectrum using a Butterworth notch filter (order = 150; low notch edge = tinnitus frequency · 2^−1/2^; high notch edge = tinnitus frequency · 2^1/2^) [16] (Figure 4). The modified training music (44100 Hz, 16 bit, stereo,.wav) was re-played with supplied portable music players (“TrekStor i.Beat move S 2.0 8 GB”, TrekStor GmbH, Lorsch, Germany) and via supplied headphones (“Sennheiser HD 201”, Sennheiser electronic GmbH & Co. KG, Wedemark Wennebostel, Germany), which are characterized by a sufficiently flat frequency response across the relevant frequency range. Patients listened to their training music in a quiet environment and were instructed to relax. It was not mandatory to focus on the training music, and patients were allowed to read or surf the internet during listening. Listening duration was 2.5 hours (without interruptions) per training day; during the first 30 min of music listening, tDCS was applied simultaneously. Patients were told not to listen to normal, non-modified music during the treatment phase.

### Tinnitus Frequency Determination

The dominant tinnitus frequency (i.e. the dominant tinnitus pitch) was matched once prior to study onset. The matching was performed by audiometrists using a clinical audiometer (Madsen Astera, GN Otometrics, Taastrup, Denmark) and a closed headphone (“Sennheiser HDA200”, Sennheiser electronic GmbH & Co. KG, Wedemark Wennebostel, Germany) following a structured protocol. The frequency resolution was 1/12 octave. In case of unilateral tinnitus, the tinnitus ear was tested. In case of bilateral tinnitus, the ear in which tinnitus was perceived as being louder was tested. In case of identical tinnitus loudness in both ears, the better hearing ear was tested.

In a first step, seven “tinnitus frequency candidates” were collected. During this procedure, the tinnitus frequency and loudness were matched seven times, starting from seven different anchor frequencies (in given order: 1000, 12500, 2000, 10000, 4000, 8000, and 6000 Hz).

In a second step, the “winner tinnitus frequency candidate” was determined. During this procedure, in each case two of the previously determined tinnitus frequency candidates (with matched tinnitus loudness) were directly compared in a two-forced-choice procedure, starting with the lowest candidate. The winner of each comparison was tested against the lowest remaining candidate frequency. This procedure was repeated until the winner tinnitus frequency candidate was found.

In a third step, an octave confusion test was performed. First, the octaves of the winner tinnitus frequency candidate between 1000 and 16000 Hz were calculated. Second, the tinnitus loudness was matched for each of these octaves. Third, the winner tinnitus frequency candidate and its octaves (with matched tinnitus loudness) were directly compared in a two-forced-choice procedure, according to step two, until the tinnitus frequency was finally determined.

### Treatment Outcome Measures

To assess treatment outcome, (i) perceived tinnitus-related distress and (ii) perceived tinnitus loudness were monitored throughout the study, i.e. prior to training onset (waiting phase), during training (treatment phase), and after training completion (observation phase) (Figure 1).

Tinnitus-related distress was assessed with (i) the Tinnitus Handicap Questionnaire (THQ; focus at perceived degree of tinnitus-related handicap) [54] as the (a priori defined) primary outcome measure, and the (ii) Tinnitus Handicap Inventory (THI; focus at perceived functioning) [55] and the German version of the Tinnitus Questionnaire (TQ; focus at tinnitus-related emotional/cognitive distress) [56] as secondary outcome measures. The THQ was defined as the primary outcome measure due to its presumed short-term change sensitivity (any number between 0 and 100 can be given as an answer to each of the items). The questionnaires were given (i) before the waiting phase, (ii) before the treatment phase, (iii) after completion of the tDCS treatment, (iv) after the treatment phase, and (v–vii) 3, 17, and 31 days after treatment completion (Figure 1). For statistical analyses, the questionnaire total scores were used. In case of the TQ, the E+C subscale (TQ_E+C_) was analyzed [16] in addition to the total score (TQ_total_).

Tinnitus loudness was estimated by visual analog scale (VAS) twice per day (scale poles: 0 ( = tinnitus gone) vs. 100 ( = personal tinnitus loudness maximum experienced so far) [16]). In the treatment phase, the loudness estimations were made directly before the beginning of the training session and 15 min after the end of the training session. During the waiting and observation phases, the loudness estimations were made with at least 4 hours in between estimations. For statistical analyses, averages across the two daily loudness estimates were used.

## Results

The data were analyzed with “Statistica 9”. The significance level was set to α = .05 (two-tailed). If the sphericity assumption of repeated measures was violated, degrees of freedom were Greenhouse-Geisser corrected. Significant main effects or interactions were further explored by means of least significant difference (Fisher LSD) post-hoc tests (family-wise error rate controlled).

### Patient Characteristics

The hearing thresholds of the patients of the three different tDCS conditions did not significantly differ (the ANOVA results showed neither a significant main effect of tDCS-CONDITION (anodal vs. cathodal vs. sham) (F_(2,28)_ = 0.38, p = 0.69) nor were there significant interactions tDCS-CONDITION×EAR (left vs. right) (F_(2,28)_ = 0.29, p = 0.75), tDCS-CONDITION×FREQUENCY (.125 vs.25 vs.5 vs. 1 vs. 2 vs. 3 vs. 4 vs. 5 vs. 6 vs. 7.1 vs. 8 vs. 10 vs. 12.5 vs. 16 kHz) (F_(26,364)_ = 0.31, p = 0.91), or tDCS-CONDITION×EAR×FREQUENCY (F_(26,364)_ = 0.71, p = 0.73)) (Figure 2). Moreover, age (F_(2,29)_ = 0.11, p = 0.90), (logarithmized) tinnitus frequency (F_(2,29)_ = 0.18, p = 0.84), and tinnitus duration (F_(2,29)_ = 1.07, p = 0.36) did not significantly differ between tDCS conditions (Table 1). Furthermore, at treatment onset (i.e. at baseline, see below) there were no significant differences in general psychopathological distress (“SCL-90-R”) [57] (F_(2,29)_ = 0.22, p = 0.81), depression (“ADS-L”) [58] (F_(2,29)_ = 1.55, p = 0.23), and state anxiety (“STAI”) [59] (F_(2,29)_ = 0.05, p = 0.95) between tDCS conditions (Table 1).

### Music Enjoyment

Enjoyment of the training music (F_(2,29)_ = 0.996, p = 0.381) as well as degree of relaxation experienced during listening to the training music (F_(2,29)_ = 0.112, p = 0.895) did not significantly differ between tDCS conditions (Table 2).

**Table 2:** Subjective music perception.

	Music perception	
Groups	Enjoyment^1^	Relaxation^1^
**Anodal tDCS** 2 **(N = 10)**	66.1 (21.2)	63.1 (22.98)
**Cathodal tDCS (N = 11)**	66.45 (20.51)	66.64 (17.25)
**Sham tDCS (N = 11)**	76.36 (15.58)	67.09 (22.51)

1 Arithmetic mean (standard deviation); range: 0–100.

2 Transcranial direct current stimulation.

### Treatment Outcome

In order to assess treatment outcome, we calculated relative change values for the time points (i) after the tDCS treatment offset at day 5 (“after tDCS + TMNMT”), (ii) after the TMNMT treatment offset at day 10 (“after TMNMT only”), (iii) three days after treatment completion at day 13 (“obs3”), and (iv) 31 days after treatment completion at day 41 (“obs31”). The baseline values were sampled directly before treatment onset. The following formula was used to calculate relative change values: [(V_(i, ii, iii, iv)_/V_baseline_)−1].

**Figure 5:**
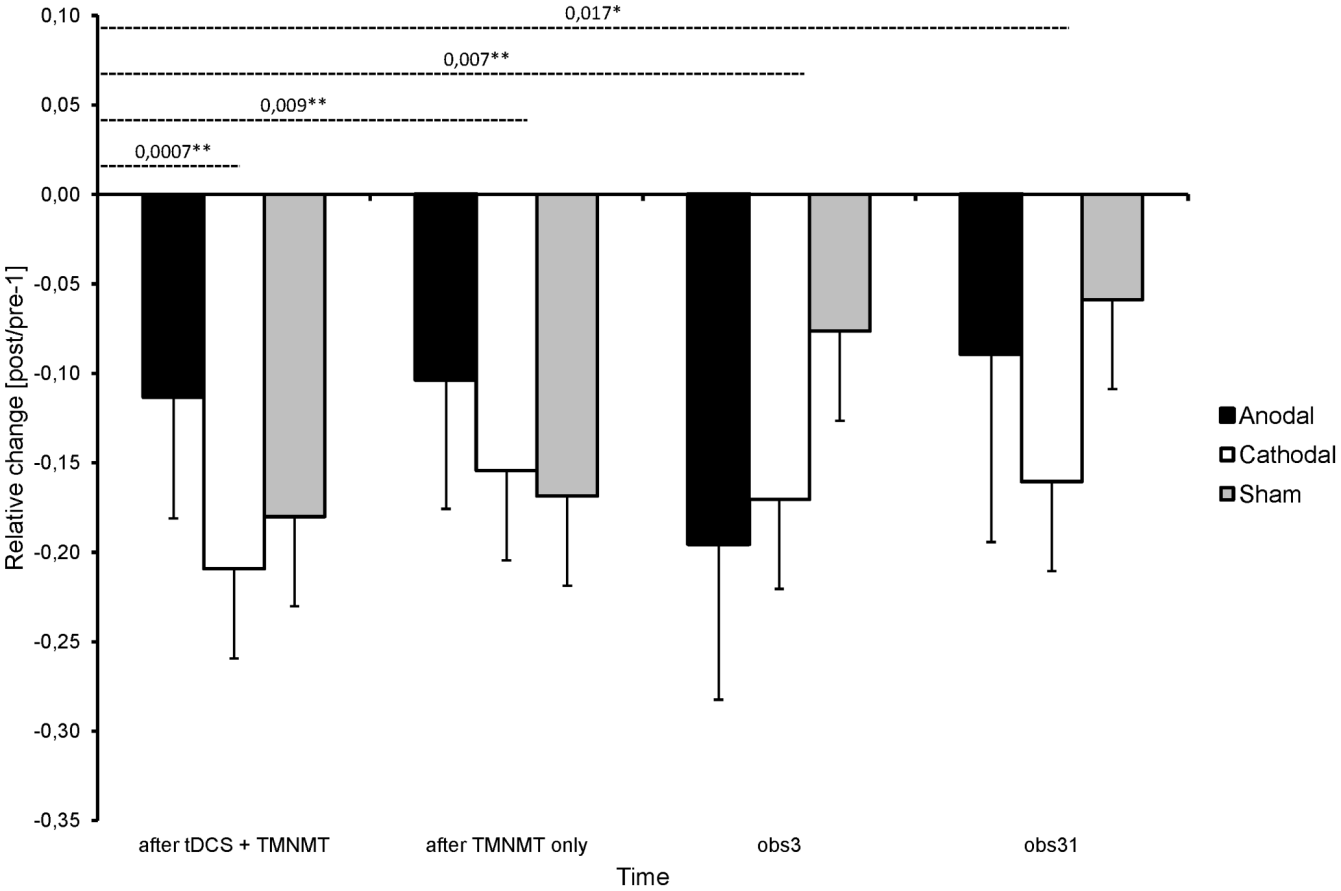
Changes in Tinnitus Handicap Questionnaire (THQ) values during and after treatment. Changes in THQ total scores relative to the baseline scores as functions of time (after tDCS^1^ + TMNMT^2^ vs. after TMNMT only vs. obs3 vs. obs31) and tDCS condition (anodal group vs. cathodal group vs. sham group). Bars represent means, error bars denote standard errors of the mean. Negative values reflect improvement. Dashed black lines indicate significant post-hoc tests. *p<.05, **p<.01; ^1^Transcranial direct current stimulation; ^2^Tailor-made notched music training.

Separately for each outcome measure (i.e. THQ change, tinnitus loudness change, TQ_total_ change, TQ_E+C_ change, and THI change), the data were analyzed by ANOVA including TIME (baseline vs. after tDCS + TMNMT vs. after TMNMT only vs. obs3 vs. obs31) as repeated measure, and tDCS-CONDITION (anodal vs. cathodal vs. sham) as between subjects measure. There was a significant main effect of TIME (F_(4,116)_ = 3.44, p = 0.042) for THQ change. Post-hoc tests revealed significant differences between “baseline” and “after tDCS + TMNMT” (p = 0.0007), “baseline” and “TMNMT only” (p = 0.009), “baseline” and “obs3” (p = 0.007), and “baseline” and “obs31” (p = 0.017) (Figure 5). There were no significant main effects or interactions for tinnitus loudness change, TQ_total_ change, TQ_E+C_ change, and THI change. The p-values for all calculated statistical tests are summarized in Table 3.

**Table 3:** Treatment outcome.

	ANOVA^1^: Main effects and interaction		
Outcomemeasures	Time	tDCS^2^condition	Time×tDCScondition
**Tinnitus** **loudness**	p = .10	p = .45	p = .76
**TQ_total_** 3	p = .56	p = .83	p = .37
**TQ_E+C_** 4	p = .16	p = .50	p = .60
**THQ** 5	p = .04*	p = .81	p = .93
**THI** 6	p = .06	p = .98	p = .91

1 Analysis of variance.

2 Transcranial direct current stimulation.

3 Tinnitus Questionnaire, total score.

4 Tinnitus Questionnaire, subscale emotional + cognitive distress.

5 Tinnitus Handicap Questionnaire, total score.

6 Tinnitus Handicap Inventory, total score.

*Statistically significant.

Given that longer lasting treatment effects would be most relevant for the efficacy of the intervention, we calculated one additional, explorative statistical test at the last time point of measurement (i.e. obs31). The one-way ANOVA using tDCS-CONDITION (anodal vs. cathodal vs. sham) as between-subjects factor did not show a significant main effect (F_(2,29)_ = 0.071, p = 0.932).

## Discussion

This study investigated whether tDCS polarity (anodal vs. cathodal) over left auditory cortex would modulate the efficacy of a combined tDCS + TMNMT short-term treatment for not severely hearing impaired patients suffering from chronic tonal tinnitus. To the best of our knowledge, this study was the first to repeatedly apply tDCS over auditory cortex in chronic tinnitus patients, and it was also the first study to combine tDCS with an acoustic neuromodulation strategy. The results indicate that, under the prevailing circumstances, there was no significant modulating effect of tDCS polarity: significant main effects or interactions of tDCS condition were neither found in the primary outcome measure (THQ; Figure 5; Table 3) nor in any of the secondary outcome measures (THI, TQ, or loudness VAS; Table 3), indicating that tDCS polarity did not influence perceived tinnitus-related distress or tinnitus loudness. However, the significant main effect of time observed in the main outcome measure (THQ; Figure 5; Table 3) implies that the combined tDCS + TMNMT short-term treatment (independently of tDCS condition) may have effectively reduced tinnitus-related distress. Alternatively, the parsimonious conclusion to draw is that this result could reflect an unspecific treatment/placebo effect. Moreover, the results of the calculated post-hoc tests, which revealed significant differences between baseline values and values at all other time points during and after treatment, but not between values at different time points during and after treatment, indicate (i) that the major efficacy component was triggered during the initial 5 days of treatment (where tDCS and TMNMT had been applied simultaneously), and (ii) that the induced reduction of tinnitus-related tinnitus distress was longer lasting, persisting beyond the end of the treatment and probably even beyond the end of the study.

tDCS appears to be a promising tinnitus treatment strategy, because this technique allows to modulate cortical excitability through anodal and cathodal stimulation, and the combination of external sensory stimulation with non-invasive brain-stimulation might enhance the effect of each stimulation by itself and increase the possibility of synaptic plasticity to occur [60]. Through tDCS, spontaneous cortical activity can be indirectly down-regulated or up-regulated, and it is assumed that synaptic transmission efficacy can be transiently altered, presumably promoting activations of neural plasticity. Thus, cathodal tDCS over auditory cortex could possibly reduce tinnitus-related hyperactivity, while anodal tDCS might either (i) boost adaptive changes triggered by treatment agents, or might (ii) change the likelihood for plastic changes to occur in the presence of other sensory input.

A study in healthy probands showed that auditory discrimination abilities can indeed decline when cathodal tDCS is applied over the surface representation of auditory cortex [53]. Surprisingly however, previous studies in chronic tinnitus patients implied that single sessions of cathodal tDCS over left temporo-parietal cortex were ineffective, while anodal stimulation could decrease perceived tinnitus intensity [38], [39]. This somewhat unexpected finding indicates that the relationship between tDCS-induced changes in cortical excitability and perceived tinnitus perception is probably more complex than theoretically predicted.

The absence of significant tDCS effects in the present study should be evaluated in the context of the specific tDCS settings that were applied. One aspect to consider is the potentially functional role of the reference electrode over right supraorbital cortex. Interactions between auditory and orbitofrontal regions could have been modulated by stimulation at either nodal point and could have had an effect on tinnitus perception. Nevertheless, there were large differences in the electrode size, making it less likely that there was a biologically meaningful effect over the orbito-frontal region. A second aspect to consider is the functional anatomy of the auditory cortex, more precisely the tonotopic organization of Heschl’s gyrus; given the usually high tinnitus frequencies, it appears conceivable that the tinnitus percept is elicited more medial than lateral on Heschl’s gyrus. A more medial location would make it harder for tDCS to have an effect on the corresponding cortex. Further, it is possible and likely that tDCS effects were not limited to the auditory cortex; rather, neural activity in associated and more remote nodes of the tinnitus network could have been enhanced or suppressed. The effects of such modulations on treatment efficacy are difficult to assess. Obviously, these points should be carefully considered in subsequent studies combining tDCS and TMNMT. Crucially, a (for instance electrophysiological) measure of cortical activity should be included in order to assess whether intended modulations of neural activity are actually achieved.

Relevant tDCS settings include for instance DC strength, stimulation duration, repetition scheme, electrode locations, and electrode sizes. In this context it appears at least unlikely that current strength (2 mA) and/or stimulation duration (30 min) were too weak or too short to evoke effects; on the contrary, compared to previous studies these parameters were rather exhausted to the limits of safe stimulation. However, optimal electrode positioning is an important point in transcranial stimulation designs. On the one hand, there are electrode location differences between different studies targeting identical cortex regions, including auditory cortex. On the other hand, modeling studies indicate that traditional electrode positioning schemes (i.e. active electrode with rather small area over the cortex region of interest, and reference electrode with rather large area far away, e.g. on the other side of the head) are probably amendable when the goal is to maximize current density in the region of interest. Moreover, differential conductivities of different brain tissues (skin, skull compacta/spongiosa, gray/white matter and in particular CSF) and corresponding local current flow differences might play an important role [23].

It is an interesting question whether and how tDCS polarity would modulate the efficacy of TMNMT. Unfortunately, the present study does not allow us to draw firm conclusions, since tDCS and TMNMT were applied simultaneously and no separate anodal or cathodal over sham effect evolved. Theoretically, tDCS and TMNMT treatments could have had interactions on different time scales. *First*, during the initial 5 days and in each case the initial 30 min of treatment, tDCS and TMNMT were applied simultaneously (Figure 1); beyond that, the TMNMT treatment was continued for another 120 min, while the tDCS treatment (which could have had lasting after-effects) was stopped. During the initial 30 min of simultaneous application, potential tDCS and TMNMT effects could have been independent from each other, complementary, or diametrical to each other, and the net effect of the combined tDCS and TMNMT treatments on tinnitus-related cortical activity could have been beneficial, detrimental, or neutral. *Secondly*, during the initial 5 days of treatment, both strategies (tDCS and TMNMT) were applied; beyond that, the TMNMT treatment was continued for another 5 days, while the tDCS treatment (which could have had lasting and accumulative effects) was stopped (Figure 1). Again, the net effect of the combined tDCS and TMNMT treatments on tinnitus-related cortical activity during the first 5 days could have been beneficial, detrimental, or neutral.

However, although it remains unresolved whether and how tDCS and TMNMT effects may have interacted, the significant post-hoc test in the main outcome measure (THQ; Figure 5; Table 3) implies that the combined tDCS + TMNMT treatment could effectively reduce tinnitus-related distress during the initial 5 days of training; this effect was independent of tDCS conditions (anodal, cathodal, and sham). Such a finding would be predicted under the following assumptions: (i) tinnitus-related cortical activity is increased for neurons coding the tinnitus frequency (“tinnitus-related peak”) compared to all other frequencies (“baseline”). (ii) TMNMT specifically reduces activity in the neurons coding the tinnitus frequency *and* increases activity in neurons coding notch edge frequencies. (iii) tDCS globally decreases/increases (depending on polarity) overall neural activity in an additive manner. (iv) The activity difference/ratio between involved (“tinnitus-related peak”) and non-involved (“baseline”) neurons would be critical for perceived tinnitus intensity, while the magnitude of baseline neural activity (i.e. activity of neurons, which are not involved into tinnitus perception) does not influence tinnitus perception. Under these conditions, a tDCS polarity-independent reduction in tinnitus intensity under combined tDCS and TMNMT treatments would be expected. In future studies, sequential combination of TMNMT and anodal/cathodal tDCS would possibly be a valuable option to treat tinnitus.

The present findings should be reviewed in the context of our previous, “pure” TMNMT studies [14]–[16], which indicated specific TMNMT efficacy. However, the present and the previous studies are difficult to compare, because there are several relevant differences between studies. Aside from parameters such as treatment duration and total listening time, which mainly differ between [14] on the one hand, and [16] and the present study on the other hand, there are also differences between [16] and the present study, such as the treatment time per day, the treatment schedule, and the treatment location. One important aspect for TMNMT efficacy may exactly be influenced by parameters such as the latter three: the patient’s perceived degrees of freedom in TMNMT execution. Degrees of freedom were maximal in [14] and [16], as patients were allowed to listen to their music whenever and wherever they wished, making it likely that the treatment was experienced as relaxing and enjoyable. Enjoyment of music is an important key factor for the activation of the reward system of the brain and for cortical plasticity [61]. In the present study however, patients had almost no degrees of freedom. Due to the intended application of tDCS, patients had to spend most of their treatment time at our institute, following a strict time schedule. The combined tDCS + TMNMT procedure may have been straining for the patients, and the load may have counteracted the positive effects of TMNMT.

## Conclusion

The present pilot study is the first attempt to simultaneously apply TMNMT and tDCS in order to treat chronic tinnitus. Our results are difficult to understand, since (i) we do not know how and where in the brain TMNMT and tDCS interact, and since (ii) the study design could have been further optimized by including additional control conditions (e.g. placebo TMNMT, wait list controls). Nevertheless, the effects in our primary outcome measure, THQ, suggest that a short-term combined treatment of tDCS + TMNMT may have reduced tinnitus-related distress. Thus, the stimulation and treatment parameters of combined tDCS + TMNMT treatment for chronic tinnitus should be further explored in future studies.
